# Delivery of fetus death with misoprostol in a pregnant woman with H7N9 avian influenza A virus pneumonia and ARDS

**DOI:** 10.1186/s13054-014-0589-7

**Published:** 2014-10-28

**Authors:** Qiang Guo, Daguo Zhao, Fenglin Dong, Shenlan Liu, Youguo Chen, Jun Jin, Dustin R Fraidenburg, Jian-an Huang

**Affiliations:** Department of Medicine, Respiratory, Emergency and Critical Care Medicine, The First Affiliated Hospital of Soochow University, 188 Shizhi st, Suzhou, 215006 China; Department of Medicine, Division of Pulmonary, Critical Care, Sleep and Allergy Medicine, University of Illinois at Chicago, Chicago, IL 60612 USA

Pregnant women are susceptible to severe pneumonia and acute respiratory distress syndrome (ARDS) from influenza, which has been associated with increased fetal loss [1]. There is very little experience for the management of pregnant women with severe pneumonia and ARDS from avian influenza A (H7N9) virus, especially when complicated by intrauterine fetal death (IUFD).

A 28-year-old pregnant woman was admitted to the hospital due to dyspnea, dry cough, and fever. The patient was at 26 weeks gestation. Infection with influenza A (H7N9) virus was confirmed from a tracheal aspirate sample using polymerase chain reaction assays. The fetus was monitored daily to check the heart rate and on admission the fetal heart rate was 140 beats/minute. The patient’s initial blood pressure was 70/45 mmHg, which was unresponsive to volume resuscitation. Norepinephrine was administrated to rescue septic shock and was required until day 11. On day 2, the beating of the fetal heart stopped as a result of severe, refractory hypoxemia (arterial oxygen pressure <50 mmHg, 24 hours). Echocardiography showed severe right ventricular dysfunction and severe left ventricular systolic dysfunction with decreased ejection fraction (26%). The renal function was slightly reduced with an elevated serum creatinine level (225 μmol/l).

Despite the administration of oseltamivir, the patient’s condition progressed quickly. Thoracic imaging showing diffuse bilateral infiltrates along with evidence of impaired gas exchange led to the diagnosis of ARDS. Difficulties in oxygenation and a deteriorating medical condition resulted in incremental positive end-expiratory pressure titrations to a maximal value of 18 cmH_2_O and plateau pressure titrations to 40 cmH_2_O. During this course, the right ventricular systolic pressure was elevated to 52 mmHg on day 12.

Severe pneumonia, septic shock, acute renal failure, acute heart failure, pulmonary hypertension, and ARDS complicated with IUFD posed a complex dilemma. On day 13, application of 200 μg misoprostol in the posterior fornix of the vagina was used to induce delivery with an induction-to-termination interval of 11.5 hours. No retained placenta or membranes were detected. Oxytocin augmentation (intramuscular injection, 10 mg) was given to prevent bleeding. In this complicated situation, misoprostol appears to be a safe, effective, and practical method for termination of IUFD [2]. Moreover, the heart function, refractory hypoxemia, pulmonary hypertension, and immunological function improved quickly on the day after the successful delivery (Table 1). Despite intensive medical therapies, catheter-related bloodstream infection and ARDS was complicated by severe pulmonary hypertension and the patient expired on hospital day 29.

**Table 1:** **Blood oxygenation, heart function, myocardial enzyme spectrum, and blood cell count data before and after the fetal death delivery in a 28-year-old woman infected with influenza A (H7N9) virus**

	**Day prior to fetal death delivery**	**Day after fetal death delivery**
**Oxygenation and respiratory data**		
PaO_2_/FiO_2_	150	187
Positive end-expiratory pressure (mmHg)	10	9
Plateau pressure (mmHg)	28	25
Lung compliance (ml/cmH_2_O)	43.4	61.5
**Basic circulation data**		
Heart rate (beats/minute)	135	114
Mean arterial blood pressure (mmHg)	105	86
Vasoactive agent	None	None
**Echocardiographic data**		
Left ventricular ejection fraction (%)	33	68
Right ventricular systolic pressure (mmHg)	52	42
Left ventricular end-diastolic diameter (mm/m^2^)	41	43
Left ventricular end-systolic diameter (mm/m^2^)	35	29
Left atrial diameter (mm/m^2^)	36	27
Interventricular septal thickness (mm/m^2^)	9	9
Left ventricular posterior wall thickness (mm/m^2^)	8	7
**Myocardial enzyme spectrum**		
Aspartate transaminase (u/l)	66	61
Creatine kinase (U/l)	377	122
Creatine kinase isomer-MB (u/l)	20	40
Lactate dehydrogenase (u/l)	448	411
Troponin (ng/ml)	0.15	0.12
**Blood cell counts**		
White blood cells (×10^9^/l)	26.7	27.4
Lymphocytes (×10^9^/l)	0.7	1.8
CD4^+^ T lymphocytes (%)	9.0	42.4
CD8^+^ T lymphocytes (%)	14.5	37.3
CD4^+^/CD8^+^	0.62	1.14
CD3^−^/CD19^+^ B lymphocytes (%)	7.7	2.3
CD3^−^CD56^+^ natural killer cells (%)	4.8	18.9

The ratio of arterial oxygen pressure (PaO_2_) to the fractional concentration of inspired oxygen (FiO_2_) (PaO_2_/FiO_2_), pulmonary dynamic compliance, heart rate, left ventricular ejection fraction, right ventricular systolic pressure, left ventricular end-systolic diameter, creatine kinase isomer-MB, number of lymphocyte cells, CD4^+^ T-lymphocyte cell count, CD8^+^ T-lymphocyte cell count and CD3^−^CD56^+^ natural killer cell count were significantly improved after the fetal death delivery.

Delivery of the fetus following IUFD using misoprostol in multiple organ failure patients was associated with improved immunological function, oxygenation, and heart function in this patient. We cannot help but consider that this may have provided more benefit early in the patient’s hospital course. Further studies may help identify the appropriate delivery time point and safety of this agent in IUFD women complicated with multiple organ failure.

## Statement

The patient’s husband provided the written informed consent for the publication in the First Affiliated Hospital of Soochow University, and this would be available for review by the Editor-in-Chief.

## Abbreviations

ARDS: Acute respiratory distress syndrome
IUFD: Intrauterine fetal death
